# Antagonistic Potential of Agro-Industrial Byproduct–Derived Lactic Acid Bacteria Against Mycotoxigenic *Aspergillus flavus* and *Fusarium verticillioides*

**DOI:** 10.1155/ijm/9002943

**Published:** 2025-11-11

**Authors:** Jannette Wen Fang Wu-Wu, Natalia Barboza, Fabián Villalta-Romero, María Viñas

**Affiliations:** ^1^Food Technology Department, Universidad de Costa Rica, San Pedro, San José, Costa Rica; ^2^National Center for Food Science and Technology (CITA), Universidad de Costa Rica, San Pedro, Costa Rica; ^3^Biotechnology Research Center, Department of Biology, Instituto Tecnológico de Costa Rica, Cartago, Costa Rica; ^4^Center for Research in Grains and Seeds (CIGRAS), Universidad Costa Rica, San Pedro, Costa Rica

**Keywords:** antifungals, lactic acid bacteria, mycotoxins

## Abstract

Mycotoxins pose significant threats to food security and human health, necessitating innovative approaches for fungal control. This study investigated the antifungal and antimycotoxigenic potential of lactic acid bacteria (LAB) isolated from agro-industrial byproducts against the toxigenic fungi *Aspergillus flavus* and *Fusarium verticillioides*. Then, 14 LAB isolates were phylogenetically characterized, revealing diverse species including *Lactiplantibacillus pentosus*, *Lactiplantibacillus plantarum, Lacticaseibacillus paracasei*, and *Leuconostoc pseudomesenteroides*. Their antagonistic activity was first screened using an overlay-streak assay, which evaluated the combined effects of competition, pH reduction, and metabolite production on mycelial growth. Subsequently, the effect of their neutralized cell-free supernatants (CFS)—containing possible pH-stable antimicrobial compounds—was tested on fungal proliferation. The results revealed a distinct, mode-of-action-dependent efficacy. In the direct coculturing assay, stronger inhibition was observed against *F. verticillioides*, with six *L. pentosus* strains achieving nearly 100% growth suppression. In contrast, CFS treatments exhibited a more pronounced inhibitory effect on *A. flavus* germination and growth rate, with *L. plantarum* 71(6)-2F showing activity comparable to a positive control. This shift in efficacy is explicitly attributed to the different mechanisms assessed in each assay: the overlay method reflects broad-spectrum inhibition driven largely by competition and acidification, to which *F. verticillioides* appears highly sensitive. The CFS assay, however, highlights the impact of specific, pH-neutral antimicrobial metabolites. Furthermore, several CFS extracts significantly reduced mycotoxin biosynthesis, suggesting these LAB metabolites can disrupt critical fungal physiological pathways. These findings underscore the potential of LAB from agro-industrial byproducts as a source of natural antifungal and antimycotoxigenic compounds.

## 1. Introduction

Mycotoxins pose a significant threat to food security by compromising the quality and safety of food at multiple levels. These toxic secondary metabolites are produced by various fungi, including *Aspergillus, Fusarium, Alternaria*, and *Penicillium* species. Mycotoxins can contaminate both food and animal feed, posing serious risks to human and animal health. Exposure to these substances can lead to a range of health problems, from mild gastrointestinal disturbances to severe liver damage, and in extreme cases, even death [1, 2]. The economic repercussions of mycotoxin contamination are severe, resulting in significant financial losses across the agricultural supply chain from crop spoilage, trade disruptions, and the high cost of compliance and safety protocols [3, 4].

The need for alternative mycotoxin control measures is increasingly urgent, as traditional methods such as chemical fungicides present challenges, including potential toxicity, regulatory restrictions, and growing fungal resistance. Additionally, chemical treatments may leave residues that are harmful to both consumers and the environment, spurring demand for natural and sustainable options [5]. Lactic acid bacteria (LAB), widely known for their role in fermentation and as probiotics, have emerged as promising biocontrol agents due to their ability to inhibit the growth of mycotoxin-producing fungi and potentially degrade or reduce mycotoxin levels [4, 6, 7]. LAB exerts their antifungal effects through several mechanisms, including the production of organic acids, bacteriocins, and other metabolites that inhibit fungal growth and mycotoxin biosynthesis. Additionally, LAB can compete with fungi for nutrients and space, further reducing the likelihood of mycotoxin production [7].

Despite the promising potential of LAB to inhibit mycotoxigenic fungi, their practical application in food and feed safety is constrained by the scarcity of robust, characterized strains for targeted use. To address this limitation, the present study was designed to source, identify, and characterize LAB strains with potential antifungal activity against major mycotoxin-producing fungi and to quantify their efficacy in reducing mycotoxin levels under controlled *in vitro* conditions. By pinpointing highly effective and versatile strains, this research seeks to develop novel, natural, and integrated strategies to improve food safety and secure food security.

## 2. Materials and Methods

### 2.1. Fungal Strains

Two mycotoxigenic fungal strains, *Aspergillus flavus* isolate 32 [8] and *Fusarium verticillioides* isolate F07 [9], were evaluated in this study. Both strains were obtained from the Mycotoxin Analysis Laboratory of the Center for Research in Grains and Seeds (CIGRAS) at the University of Costa Rica (UCR).

For inoculum preparation, Petri dishes prepoured with Czapek yeast extract agar (PhytoTech Labs, United States) were inoculated with 10 *μ*L of a spore solution placed at the center of each plate. The plates were then incubated for a minimum of 5 days at 26°C ± 0.5°C for *F. verticillioides* and at 30°C ± 0.5°C for *A. flavus*. Following incubation, the plates were scraped twice using 1 mL of sterile water to prepare a spore suspension. The concentration of spores in the suspension was determined by microscopy to ensure a minimum spore load of 10^6^ spores per microliter.

### 2.2. LAB Strains

Then, 14 LAB isolates previously obtained from agro-industrial byproducts [10, 11], along with two commercial reference strains, were used in this research. All isolates were supplied by the Microbiology Laboratory of the National Center for Food Science and Technology (CITA) at the UCR. The bacterial strains were cultivated in Man-Rogosa-Sharpe (MRS) broth (Oxoid, Basingstoke, United Kingdom) under capnophilic conditions at 35.0°*C* ± 0.5°*C* for a minimum of 24 ± 2 h. These conditions were maintained to ensure optimal bacterial growth and viability prior to their application in the experimental assays.

### 2.3. LAB Identification

LAB were grown in MRS agar (Oxoid, Basingstoke, United Kingdom) for 22 ± 2 h at 35.0°*C* ± 0.5°*C*. Total nucleic acids were extracted from each isolate using a miniprep extraction protocol [12]. The primer pair 27F/1492R was used to obtain a 1.5-kb fragment of the 16S rRNA gene [13] and PCR was conducted according to Wu et al. [11]. The sequences obtained were aligned using the Basic Local Alignment Search Tool (BLAST) provided by the National Center for Biotechnology Information (NCBI, Bethesda, MD, United States) within the rRNA/ITS database. A search was specifically conducted for 16S ribosomal RNA sequences. Identifications with a match percentage of 97% or greater were considered the most accurate at the species level.

Sequence alignments were performed using MEGA X [14] with the MUSCLE algorithm [15]. Phylogenetic analysis was conducted using Bayesian inference analysis with four chains, each run for 10 million generations with a burn-in of 25%.

### 2.4. Screening of LAB Antagonist Capacity Against Mycotoxigenic Fungi

The determination of the antagonistic capacity of each LAB was conducted by evaluating microbial activity using the overlay streak method, based on the methodologies of Pektas [16] and Russo et al. [17]. All the studied LAB were evaluated against *F. verticillioides* and *A. flavus*. The LAB were streaked on MRS agar (Oxoid, Basingstoke, United Kingdom) in a thick, straight line approximately 7-cm long, with a 0.5-cm distance from the edge of the plate. The plates were incubated under capnophilic conditions at 35.0°*C* ± 0.5°*C* for 24 ± 2 h. After this period, an overlay of approximately 10 mL of Czapek (Phytotech Labs, United States) with yeast extract agar (Sigma-Aldrich, Merck Group, United States) was applied. This overlay was inoculated with 10 *μ*L of a spore solution containing 10^6^ spores per microliter of the fungus to be evaluated. The plates were then incubated for 7 days at 26°*C* ± 0.5°*C* for *F. verticillioides* and at 30°*C* ± 0.5°*C* for *A. flavus*. Triplicate biological replicates of each LAB strain were prepared, alongside a control plate inoculated solely with the fungal pathogen. The antagonistic effect was assessed by measuring and comparing the size of the fungal colonies using Equation (1).

(1) $$\begin{array}{c} \text{Fungal growth inhibition}\left(\%\right)=\frac{\left(\text{Dc}-\text{Dt}\right)}{\text{Dc}}\times 100, \end{array}$$

where Dc is the diameter of the fungal colony in the control and Dt is the diameter of the fungal colony when LAB is present.

### 2.5. Determination of Cell-Free Supernatant (CFS) Inhibitory Effect on Fungal Growth

The inhibitory effect of the supernatant on the fungi was determined using the strains that showed the most promising results in the previous screening. The LAB was grown in MRS broth at 35°C ± 0.5°C for 24 ± 2 h. After this period, the culture was centrifuged at 1500 rpm for 15 min. The supernatant was then decanted and filtered through a 0.2-*μ*m filter to remove any residual cells from the medium. The supernatant was neutralized with 0.1 M NaOH and used immediately to eliminate interference from lactic acid present in the media.

To evaluate the effect of LAB-CFS on fungal growth and mycotoxin production, an assay was performed using the 96-microwell plates. Each well was filled with a mixture of potato dextrose broth (PDB, Oxoid, Basingstoke, United Kingdom), peptone water 0.1% (Oxoid, Basingstoke, United Kingdom), CFS in a 1:1:1 ratio, and fungal spores (10^6^ spores/*μ*L) for a total volume of 200 *μ*L per well. For the controls, the same composition as previously mentioned was used with some modifications: in the negative control, supernatant was substituted with sterile MRS broth (Oxoid, Basingstoke, United Kingdom), while the positive control included sterile MRS spiked with 0.1% cycloheximide for a final concentration of 0.025% *μ*L (*v*/*v*) in 200 *μ*L (Oxoid, Basingstoke, United Kingdom; a media control). Three control treatments were included: a negative control, identical to the test mixture, but with CFS replaced by sterile MRS broth (Oxoid, Basingstoke, United Kingdom); a positive control, sterile MRS broth spiked with 0.1% cycloheximide to achieve a final concentration of 0.025% (*v*/*v*) in the 200 *μ*L well; and a media control, malt broth (Oxoid, Basingstoke, United Kingdom) for *F. verticillioides* and yeast-glucose broth (Oxoid, Basingstoke, United Kingdom) for *A. flavus*.

The plates were incubated for 48 h at 26°*C* ± 0.5°*C* for *F. verticillioides* and 30°*C* ± 0.5°*C* for *A. flavus*. Fungal growth kinetics were monitored by measuring the optical density at 550 nm every 24 h using a Multiskan Go spectrophotometer (Thermo Scientific, United States).

Following incubation, the entire volume from corresponding wells was pooled, transferred to 2-mL microcentrifuge tubes, and centrifuged at 14,000 × *g* for 10 min. The supernatant was then filtered through a 0.2-*μ*m membrane into amber glass vials and stored at −20°C until subsequent mycotoxin analysis.

### 2.6. Mycotoxin Quantification Through UHPLC-MS

Mycotoxin analysis was performed by ultra-high-performance liquid chromatography coupled with tandem mass spectrometry (UHPLC-MS/MS) using an adapted method based on León-Cortés et al. [18] and Viñas et al. [19].

The analysis was conducted on a UHPLC Ultimate 3000 system (Thermo Scientific, United States) coupled to a TSQ Endura triple quadrupole mass spectrometer (Thermo Fisher Scientific, MA, United States) and controlled by TraceFinder 3.2 quan software. Chromatographic separation was achieved using a C18 analytical column (Accucore aQ, 100 × 2.1 *mm*, 2.6 *μ*m) protected by a corresponding guard column (10 × 2.1 *mm*, 2.6 *μ*m) (Thermo Fisher Scientific, Pittsburgh, United States), both maintained at 45°C. The mobile phase flowed at 400 *μ*L/min and consisted of (A) water/MeOH/formic acid (97:2:1, *v*/*v*/*v*) and (B) MeOH/water/formic acid (97:2:1, *v*/*v*/*v*). The gradient elution program was as follows: 5% B at 0 min, increased linearly to 50% B at 3 min, 75% B at 6 min, and 100% B at 8 min, before re-equilibrating at 5% B from 11.0 to 11.1 min, yielding a total run time of 11.1 min. The injection volume was 5 *μ*L, and the autosampler temperature was maintained at 10°C.

Mass spectrometric detection was performed using a heated electrospray ionization (H-ESI) source in positive ionization mode with selected reaction monitoring (SRM). The source parameters were optimized as follows: spray voltage, 3500 V; sheath gas, 50 (arbitrary units); auxiliary gas, 10 (arbitrary units); sweep gas, 1 (arbitrary unit); ion transfer tube temperature, 342°C; and vaporizer temperature, 358°C. Argon was used as the collision gas at a pressure of 1.5 mTorr. The cycle time was set to 0.5 s.

### 2.7. Statistical Analysis

Two-tailed Student's *t*-test with unequal variances (*alpha* = 0.05) was used to compare growth rates and mycotoxin levels between treatment groups and the negative control. Mean values and standard deviations for each group were calculated from triplicate experiments to assess variability within treatments. All analyses were performed using Microsoft Excel and JMP18 software (SAS Institute, Cary, NC).

## 3. Results

### 3.1. LAB Identification and Phylogenetic Analysis

**Figure 1:**
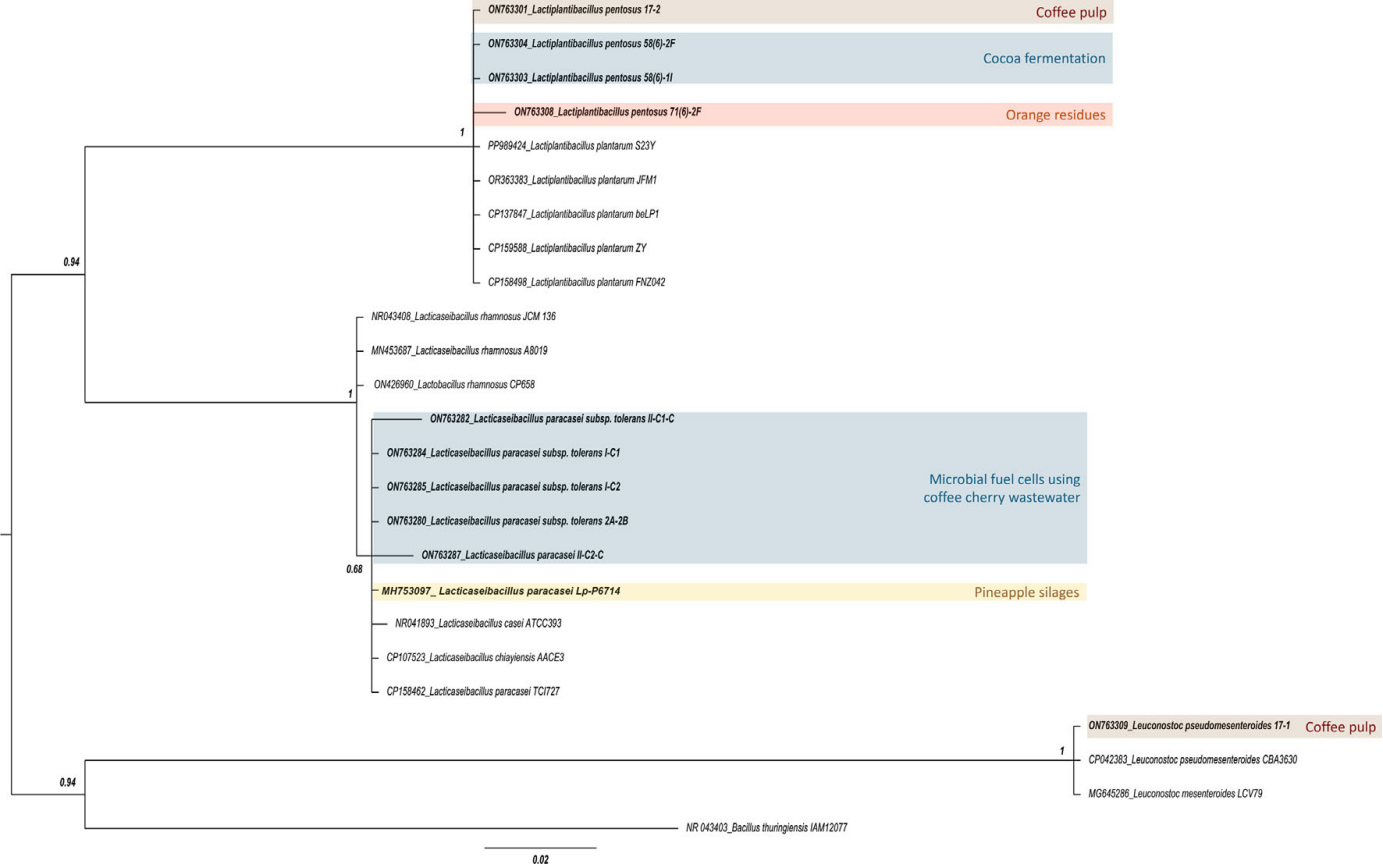
Bayesian phylogenetic tree of *lactic acid bacteria* strains isolated from various agro-industrial byproducts, inferred using MrBayes. Posterior probability values are shown at key nodes. The tree is rooted using *Bacillus thuringiensis* IAM12077 as an outgroup. Highlighted isolates show the agro-industrial byproduct from where each one was obtained.

The phylogenetic analysis of the LAB isolates (Figure 1) provides insights into the taxonomic relationships among the strains. The analysis reveals distinct clades, indicating differentiation at the species level for some of the isolates.

Phylogenetic analysis revealed a prominent clade of *Lactiplantibacillus pentosus* and *L. plantarum*, species whose close relationship suggests similar adaptation to vegetable niches. These strains have previously been isolated from cocoa fermentation, coffee waste, and oranges [20–22] which suggests their presence as native microbiota responsible for their fermentative processes. The second clade, grouping *Lacticaseibacillus paracasei, L. casei*, and *L. rhamnosus*, was identified in coffee waste and pineapple-fermented waste. Although more commonly found on dairy products, they have also been reported on plants (sources [11]). *Leuconostoc pseudomesenteroides* formed a distinct phylogenetic clade separate from the *Lactiplantibacillus* and *Lacticaseibacillus* genera, which is consistent with its presence in spontaneous cocoa bean (fermentations [23]).

### 3.2. Inhibitory Effect of LAB Against Fungi

**Figure 2:**
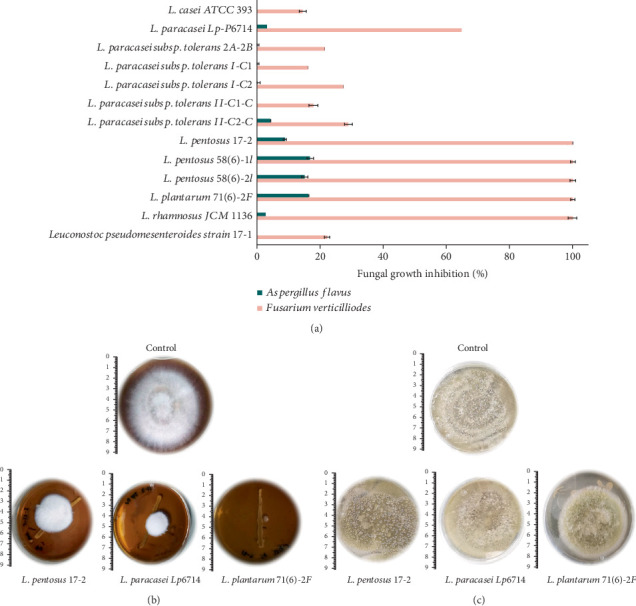
Antifungal screening of lactic acid bacteria (LAB): Fungal growth inhibition measured as reduction of the mycelium diameter of *Aspergillus flavus* and *Fusarium verticillioides* by LAB isolates through overlay-streak assays: (a) Percentage of fungal growth inhibition, (b) pictures showing the colony growth of *F. verticillioides*, and (c) *A. flavus* in the presence of LAB.

The antifungal activity of various LAB strains against the mycotoxigenic fungi *Aspergillus flavus* and *Fusarium verticillioides* indicates significant strain-specific differences in the inhibition of fungal growth shown as a reduction of the mycelium diameter (Figure 2).

Overall, the LAB strains demonstrated a more pronounced inhibitory effect against *F. verticillioides* compared to *A. flavus*. Six LAB isolates, particularly *L. pentosus* strains, exhibited nearly 100% inhibition of *F. verticillioides* growth. In contrast, the antifungal activity against *A. flavu*s was much lower, with only a few *L. pentosus* isolates showing moderate inhibition of around *15%–17%.* In general, most of *L. paracasei* and *L. casei* showed the least inhibitory effect, particularly against *A. flavus*.

### 3.3. Effect of LAB-CFS on Fungal Growth Rate and Mycotoxin Production

**Figure 3:**
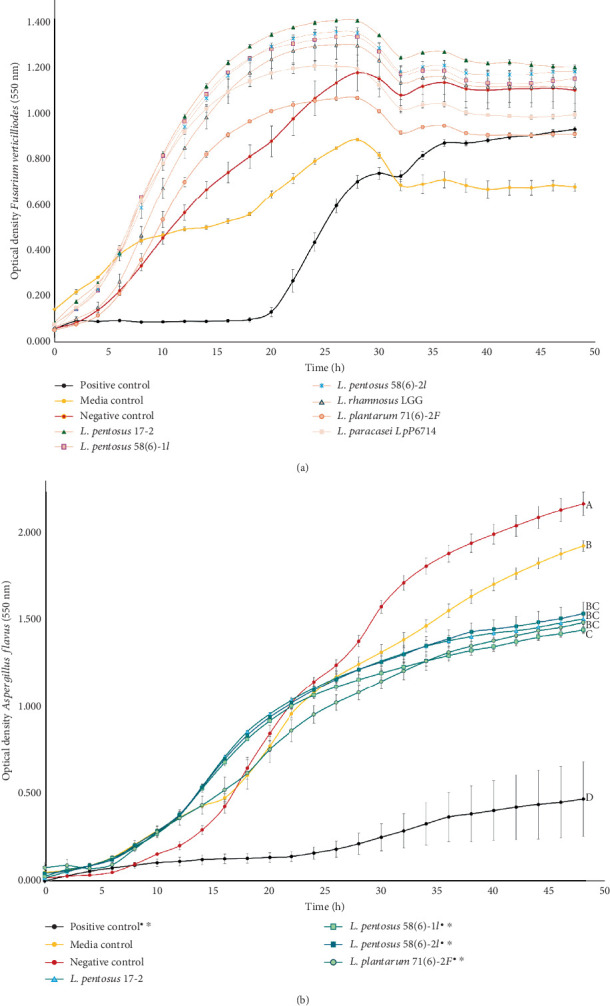
Impact of lactic acid bacteria cell-free supernatants (LAB-CFS) on fungal growth kinetics. Growth curves of (a) *Fusarium verticillioides* and (b) *Aspergillus flavus* in media supplemented by various LAB-CFS treatments. Values are expressed as *mean* ± *standard* *error* (*n* = 3). Treatments labeled with different letters exhibit statistically significant differences in growth rate by Tukey's HSD (*α* = 0.05). Significant differences from the negative control and media control for individual time points (analyzed by a two-tailed *t*-test) are indicated by (∗) and (•), respectively.

The inhibitory effect of LAB-CFS on the growth of mycotoxigenic fungi and mycotoxin production was evaluated over a 48-h period. The application of LAB-CFS significantly altered the growth kinetics of both *F. verticillioides* (Figure 3a) and *A. flavus* (Figure 3b) compared to the negative and media controls.

The growth rate, derived from the slope of the exponential phase, differed significantly from the controls for most treatments (one-way ANOVA, Tukey's HSD, *p* < 0.05). Surprisingly, for *F. verticillioides*, all LAB isolates elicited a significant increase in growth rate compared to the media control. A similar stimulatory effect was observed relative to the negative control, except for L. *plantarum 71(6)-2F* and *L. paracasei* LpP6714, which showed no significant difference in growth rate. It is noted that the population in these latter treatments declined after entering the stationary phase, indicating a potential late-stage inhibitory effect not captured by the growth rate alone.

In contrast, the growth of *A. flavus* was significantly inhibited by all LAB isolates. The growth rate was significantly reduced compared to both the media and negative controls. This inhibitory effect was particularly pronounced during the later stages of growth (30–48 h), as evidenced by a substantially lower optical density in the treatment groups.

Growth curves for the negative control (sterile MRS broth) and the media control displayed standard sigmoidal kinetics, entering exponential growth rapidly and achieving a high cell density in the stationary phase. A notable exception was observed for *F. verticillioides* (Figure 3a), where growth in the media control was significantly attenuated. Statistical comparison of the growth rates confirmed that the negative control supported a significantly higher rate of growth for both *F. verticillioides* and *A. flavus* compared to their respective media controls.

All tested CFS completely suppressed the production of both fumonisin B₁ (FB1) and aflatoxin B₁ (AFB1) to levels below the detectable limit. In stark contrast, significant mycotoxin concentrations were measured in the control groups. The fungus cultivated without CFS (negative control) produced 3.3 ± 0.4* μ*g/kg of FB1 and 7.2 ± 0.6* μ*g/kg of AFB1. In contrast, the fungus grown in the media control showed a production of 8.6 ± 0.6* μ*g/kg of FB1, but a lower concentration of AFB1 (4.9 ± 0.97* μ*g/kg). The fungus cultivated with the 0.025% cycloheximide (positive control) resulted in no detectable FB1 (ND), while AFB1 levels (7.5 ± 0.8* μ*g/kg) were comparable to those in the negative control.

Statistical analysis confirmed that the mycotoxin concentrations in all LAB-CFS treatments were significantly different from those in the control groups (*p* < 0.05). The results demonstrate that the LAB-CFS from all six strains exhibited a strong antimycotoxigenic effect, effectively preventing the synthesis of both major mycotoxins under experimental conditions.

## 4. Discussion

LAB plays a crucial role in diverse natural environments and food systems due to their ability to produce a wide range of metabolites with potential industrial and medical applications. These include antimicrobial compounds such as organic acids, bacteriocins, and hydrogen peroxide, which effectively inhibit the growth of various harmful microorganisms [24]. However, the effectiveness and functional properties of LAB can vary significantly across strains, particularly when considering autochthonous populations that have adapted to specific local environments [25, 26].

To better understand the relationships between LAB from indigenous sources, a phylogenetic analysis of several autochthonous strains was conducted using the Bayesian inference algorithm method (Figure 1). As noted before, the phylogenetic analysis revealed consistent patterns of relationships among the LAB isolates. The occurrence of these bacteria in food byproducts and food is relevant, as they play crucial roles in fermentation and food preservation. Studies have highlighted their presence in diverse food matrices, demonstrating their adaptability and functional diversity, which offer possibilities for different industrial applications [27–29].

Members of the *Lactobacillus* genus thrive in diverse environments characterized by high nutrient availability. These bacteria are prevalent in both deteriorating and intentionally fermented food products, while also colonizing agricultural animal feeds and soil ecosystems. Additionally, they are natural inhabitants of the gastrointestinal tracts of both vertebrate and invertebrate organisms [30, 31]. The presence of *L. pentosus, L. plantarum*, and *L. paracasei* in fermented foods such as cocoa fermentation and pineapple silages suggests their robust adaptability to various nutrient matrices and fermentation conditions. *L. pentosus* shows a preference for plant-derived substrates [32], while *L. paracasei* exhibits a stronger association with dairy-based environments, likely due to its enhanced ability to metabolize lactose and milk proteins [33, 34]; however, it has also been reported in plant-based niches. Some studies have reported their presence in diverse fermentative processes such as cocoa bean [35] and coffee bean production [36] in which they play a regulatory and sensory quality role. This distribution pattern aligns with genetic analyses suggesting evolutionary adaptation to specific ecological niches [34].

*Leuconostoc* sp. has also been reported as a recurrent microorganism in both dairy and plant-based sources and can frequently be found in vegetables, cereals, silages, fruits, wine, fish, meat, and dairy products [37]. Unlike *L. plantarum*, *L. pentosus*, and *L. paracasei*, *L. pseudomesenteroides* has often been associated with food spoilage [38]. However, in this work, this species was found in coffee pulp, where it might play an active role during coffee fermentation. *Leuconostoc* sp. and other LAB are naturally present in fresh coffee cherries. During the depulping process, which separates coffee beans from pulp, *Leuconostoc* sp. proliferates in the mucilage area and becomes the predominant bacterial species in the early fermentation stages [39]. Studies regarding their use as potential probiotics or bacteriocin sources have been limited; however, their capacity to produce dextran has gained interest for industrial applications [37].

While 16S rRNA gene sequencing has emerged as a powerful tool for the identification and taxonomic classification of bacteria, this approach is not without its limitations. The high degree of sequence conservation within the 16S rRNA gene can obscure meaningful genetic differences between closely related bacterial species or even strains, leading to potential misidentification or over-simplification of microbial diversity due to limited sequence variation [40]. To overcome these limitations, complementary approaches incorporating whole-genome sequencing, targeted metabolic profiling, and advanced bioinformatic analyses might be essential in achieving a more comprehensive understanding of the taxonomic structure.

zRegarding the inhibitory effects of the LAB strains on the growth of *A. flavus* and *F. verticillioides*. The findings revealed notable fungal growth inhibition, which is critical for understanding the potential of these bacterial strains as biocontrol agents. As shown in Figure 2a, the chosen LAB strains demonstrated a differentiated inhibitory effect on the growth of both *F. verticillioides* and *A. flavus.* Overall, the inhibitory effect was more significant on *F.* verticillioides than on *A. flavus.* Many of the LAB isolates showed moderate to high inhibition against *F. verticillioides* but negligible impact on *A. flavus*. Six isolates, in particular *L. plantarum 71(6)-2F*, *L. pentosus* 58(6)-2I, 58(6)-1I and 17-2, *L. paracasei LpP6714*, and *L. rhamnosus* LGG exhibited higher levels of inhibition against *F. verticillioides*, suggesting a strong antagonistic effect against this fungal species that may be attributed to the presence of antifungal metabolites as well as stress competition. These strains are characterized by high lactic acid production that causes a significant decrease in pH. Literature reports that *Fusarium* species have an optimal growth at pH 6, and their growth rate was significantly reduced at lower pH values (*pH* ≥ 3) [41]. Previous studies have shown that in optimal conditions these LAB strains could achieve pH 2 or lower, inducing stressful conditions for the fungi [11, 42].

Bacteria with the highest antagonistic effect against *F. verticillioides* were then evaluated against *A. flavus.* However, their effect on this fungus was much weaker, indicating a potential species-specific response. Only *L. pentosus* and *L. plantarum* isolates showed an effect, albeit low (15%–17%), against this fungus.

The observed antifungal inhibition is likely multifactorial. Potential mechanisms include direct competition for nutrients and space, as well as the production of fungus-specific antimicrobial compounds, such as bacteriocins. A significant contributing factor is the production of organic acids by LAB, which acidifies the growth medium and creates an unfavorable environment for fungal development [43]. The differential efficacy against the two fungi suggests that each bacterial strain employs a distinct combination of these mechanisms, with their relative importance depending on the specific fungal target. This could be attributed to differences in cell wall structure or metabolic pathways between the two fungal species, which might affect how susceptible they are to the metabolites produced by LAB [43, 44]. The cell wall compositions of *Aspergillus* and *Fusarium* differ significantly, affecting their susceptibility to antimicrobial agents. In *Aspergillus*, the cell wall is primarily made up of chitin, *β*-glucan, and *α*-glucan, forming a rigid core structure that contributes to its growth and virulence—this is referred to as the “rigid phase.” Additionally, the presence of galactomannan and galactosaminogalactan in the “mobile phase” allows *Aspergillus* to adapt dynamically to environmental stresses, including exposure to antimicrobial compounds [45]. In contrast, *Fusarium* species possess a higher chitin content and a distinct polysaccharide organization. The variation in glucan composition across species affects cell wall elasticity and alters susceptibility to antimicrobial agents, while also contributing to increased virulence. Additionally, smaller components like melanin and galactomannans influence cell wall pigmentation and enhance resistance to environmental stressors [46].

At this stage, the results were significantly affected by the presence of viable LAB cells, highlighting a key limitation of the technique: its dependence on co-culturing. The different growth rates of fungi and bacteria create a competitive environment that could skew the observed results [47, 48]. Given their higher metabolic efficiency, bacteria have a distinct advantage in growth speed, potentially depleting available nutrients and leaving less for fungal exploitation. Although the results demonstrate a reduction in fungal growth, this does not necessarily indicate that the inhibition is due to the production of bioactive metabolites of interest [49, 50]. Therefore, analyzing the effects of the CFS is a crucial assay, as it helps distinguish between inhibition due to growth competition and acid-mediated suppression. Based on the results observed on the co-culturing assay (Figure 2), strains that demonstrated promising inhibitory effects against *F. verticillioides* (two LAB strains) and *A. flavus* (four LAB isolates) were evaluated (Figure 3). The positive control containing a known antifungal agent (0.025% cycloheximide) effectively inhibited both fungal species, confirming the experimental setup's ability to assess antifungal activity (Figure 3).

Analysis of the extended growth kinetics revealed distinct, strain-specific responses to LAB-CFS treatments, underscoring the complex interplay between fungal pathogens and bacterial metabolites. Particularly for *F. verticillioides*, the application of certain LAB-CFS treatments appeared to stimulate fungal growth, resulting in a higher optical density than both the negative and media controls. This promotive effect is likely attributable to the nutritional composition of the supernatants. Unlike the media control (malt extract-based, rich in carbohydrates but limited in nitrogen, designed for fungal cultivation), the LAB-CFS contained spent MRS broth. MRS is a complex source of peptides, amino acids, and vitamins that were premetabolized by LAB. LAB possess a sophisticated proteolytic system that hydrolyzes proteins into readily assimilable amino acids, enhancing the bioavailability of nitrogenous nutrients for the fungus and potentially explaining the observed growth stimulation [51].

The specific amino acid profile in the CFS could influence this response. The effect of amino acids on *Fusarium* is complex and species-specific; for instance, while aspartic acid, methionine, and cysteine can inhibit growth but stimulate mycotoxin production, others like glutamine and arginine may promote overall fungal growth and metabolism [52]. Therefore, the presence of specific free amino acids derived from LAB proteolysis could have inadvertently acted as growth promoters for *F. verticillioides* in this system as seen in kinetics (Figure 3a). The quantification of FB1 production revealed stark contrasts between treatments. FB1 was produced at higher levels in media control compared to the negative control and was below the detection limit in all LAB-CFS treatments. This differential production indicates that while the nutritional composition of the growth medium (e.g., the malt-based media control) is a primary driver of mycotoxin synthesis, it does not fully explain the absence of FB1 in the CFS treatments. The fact that FB1 was still detectable in the nutrient-rich negative control suggests that the LAB-CFS contains active metabolite(s) that directly inhibit the biosynthetic pathway of FB1, surpassing the baseline level of suppression offered by nutrient competition alone.

In contrast to *F. verticillioides*, the growth of *A. flavus* was significantly inhibited by all LAB-CFS treatments. The fungus thrived in the negative and media controls (yeast-glucose broth) but showed substantially reduced proliferation in the presence of LAB supernatants, particularly that of *L. pentosus* 71 (Figure 3b). The antifungal activity of the LAB-CFS was accompanied by a complete inhibition of AFB1 synthesis, confirming a potent antimycotoxigenic effect that extended beyond mere fungistasis (Table 1). While AFB1 was produced in all control groups, it remained nondetectable in all CFS-treated cultures.

Paradoxically, the highest AFB1 accumulation coincided with the lowest fungal biomass in the cycloheximide-treated positive control. Although counterintuitive, this result aligns with early literature reports that cycloheximide can promote aflatoxigenesis. The mechanism is thought to involve induced metabolic stress or the disruption of aflatoxin regulatory networks via inhibition of key protein synthesis [53, 54]. This is consistent with the foundational work of Buchanan et al. [55], who demonstrated that cycloheximide, a translation inhibitor, has a time-dependent effect on aflatoxin production in *A. parasiticus*. Their study demonstrated that cycloheximide addition before 6 h post-induction prevented aflatoxin synthesis, while later addition resulted in increasing toxin yields. In our experiment, however, cycloheximide was added at 0 h upon inoculation with spores from a pre-cultivated plate. Based on the mechanism proposed by Buchanan et al., this early addition should have prevented AFB1 production. Paradoxically, we observed the opposite effects. This discrepancy may be attributed to critical differences in experimental conditions. The composition of the growth media might be a key factor, as the negative and positive controls quantified similar AFB1 content. Other variables, such as the fungal species (*A. flavus* vs. *A. parasiticus*), the physiological state of the inoculum (spores vs. mycelia), or subtle differences in cycloheximide concentration, could also explain the contrasting results. Overall, the CFS exhibited a stronger inhibitory effect against *A. flavus* than against *F. verticillioides*. Some metabolites potentially produced by LAB such as antimicrobial peptides (AMPs) have also been shown to inhibit conidial germination through the inhibition of germ tube elongation after conidial wall breakdown [56, 57]. These compounds may also modulate gene expression leading to a decreased viability of fungal cells. In other instances, metabolites such as bacteriocins and bacteriocin-like substances (BLISs) have been shown to be able to inhibit the growth of fungal cells by blocking the expression of certain genes or by modulating *quorum sensing* [58]. Other metabolites including organic acids, carboxylic acids, phenolic acids, cyclic dipeptides, hydrogen peroxide, reuterin, peptides, and miscellaneous antifungal compounds had also been reported to inhibit a broad spectrum of fungal species [43, 58, 59]. This variety of antimicrobial metabolites may impact directly on genus-specific metabolisms, leading to different responses by the two fungi.

The antimycotoxigenic mechanism may operate through two distinct pathways: (1) the direct suppression of mycotoxin biosynthesis (mycotoxigenesis) within the fungus, or (2) the enzymatic degradation of mycotoxins already present in the medium, facilitated by extracellular enzymes secreted by the LAB into the CFS such as proteases, esterases, and peptidases [60]. However, for the enzymatic degradation pathway to be considered a truly effective and safe detoxification strategy, future research must focus on identifying the resulting degradation products and rigorously assessing their toxicity, as some metabolites could potentially be more hazardous than the parent mycotoxin [61].

Further studies should focus on isolating individual inhibitory compounds for a more precise assessment. Although results are not conclusive, these suggest that the presence of CFS may disrupt fungal physiology, likely affecting mycotoxin production. Mycotoxin biosynthesis is highly responsive to environmental stresses, which activate signal transduction pathways that induce production. Key factors, including temperature, humidity, and nutrient availability, are crucial in regulating the transcription of genes involved in mycotoxin biosynthesis [62]. Inhibitory compounds can significantly reduce mycotoxin production in fungi by disrupting various stages of fungal growth and biosynthesis. These compounds may act directly as inhibitors or as signaling molecules that block the expression of genes associated with mycotoxin production, ultimately leading to reduced toxin levels [27, 63, 64].

However, the use of CFS is limited, primarily due to the naturally low concentrations of antimicrobial compounds and the variability introduced by environmental factors that may affect their stability and effectiveness [65]. Nevertheless, these results show the potential of these compounds as an effective measure in mitigating fungal contamination and enhancing food safety.

## 5. Conclusions

This study highlights the antifungal and antimycotoxigenic potential of LAB isolated from agro-industrial byproducts, particularly in inhibiting mycotoxigenic fungi such as *A. flavus* and *F. verticillioides*. The findings demonstrate strain-specific inhibition, with several LAB strains effectively suppressing fungal growth and potentially reducing mycotoxin production. The CFS from LAB showed significant initial inhibitory effects on fungal growth, suggesting that the depletion or degradation of active compounds may limit sustained inhibition.

Additionally, this research underscores the need for advanced phylogenetic analysis to better understand the genetic diversity and potential antifungal mechanisms of LAB isolates. Through co-culturing assays and CFS tests, this study provides insights into the mechanisms of fungal inhibition, suggesting that LAB-derived compounds act either by direct inhibition or by disrupting signal pathways that regulate mycotoxin biosynthesis.

These findings highlight the potential of the LAB as a biological control and biodetoxification agent against these harmful fungal organisms and mycotoxins. Future research should focus on identifying and optimizing active compounds within LAB-CFS to enhance the stability and persistence of their antifungal effects. These findings show the potential of LAB as natural biocontrol agents, offering a promising strategy for mitigating mycotoxin contamination and promoting sustainable food preservation practices. Further studies are needed to clarify the specific mechanisms underlying LAB antifungal and antimycotoxin activity and to assess their practical applications across agricultural and food systems.

## Figures and Tables

**Table 1:** Concentration of mycotoxins produced by *Fusarium verticillioides* and *Aspergillus flavus* after 48 h incubation with lactic acid bacteria cell-free supernatant (LAB-CFS). Values represent *mean* *concentration* (*μg*/*mL*) ± *standard* *deviation* (*n* = 3). Within each mycotoxin, least-squares means followed by different letters are significantly different (*p* < 0.05).

**Sample**	**Mycotoxin concentration (*μ*g/kg)**	
	**Fumonisin B1**	**Aflatoxin B1**
Negative control	3.3 ± 0.4^b^	7.2 ± 0.6^A^
Positive control	ND	7.5 ± 0.8^A^
Media control	8.6 ± 0.6^a^	4.9 ± 0.97^B^
*L. pentosus* 17-2	ND	ND
*L*. *pentosus* 58 (6)-1I	ND	ND
*L*. *pentosus* 58 (6)-2I	ND	ND
*L*. *rhamnosus LGG*	ND	ND
*L. paracasei* LpP6714	ND	ND
*L. plantarum* 71 (6)-2F	ND	ND

*Note:* ND denotes that the mycotoxin concentration was below the limit of detection.

^a^ and ^b^ denote *p* = 0.0001.

^A^ and ^B^ denote *p* = 0.0007.

## Data Availability

Data underlying the results of this study is not publicly available but may be obtained from the corresponding author upon reasonable request.

## Nomenclature

LAB: Lactic acid bacteria
MRS: De Man, Rogosa, and Sharpe
CFS: Cell-free supernatant
UCR: University of Costa Rica
